# Sequential above- and belowground herbivory modifies plant responses depending on herbivore identity

**DOI:** 10.1186/s12898-017-0115-2

**Published:** 2017-02-08

**Authors:** Dinesh Kafle, Anne Hänel, Tobias Lortzing, Anke Steppuhn, Susanne Wurst

**Affiliations:** 1 Functional Biodiversity, Dahlem Centre of Plant Sciences, Institute of Biology, Freie Universität Berlin, Königin-Luise-Str. 1-3, 14195 Berlin, Germany; 2 Molecular Ecology, Dahlem Centre of Plant Sciences, Freie Universität Berlin, Haderslebener Str. 9, 12163 Berlin, Germany

**Keywords:** Above- and belowground interaction, Induced plant defense, Priming, Feeding guilds, Resistance, Tolerance

## Abstract

**Background:**

Herbivore-induced changes in plant traits can cause indirect interactions between spatially and/or temporally separated herbivores that share the same host plant. Feeding modes of the herbivores is one of the major factors that influence the outcome of such interactions. Here, we tested whether the effects of transient aboveground herbivory for seven days by herbivores of different feeding guilds on tomato plants (*Solanum lycopersicum*) alters their interaction with spatially as well as temporally separated belowground herbivores.

**Results:**

The transient aboveground herbivory by both chewing caterpillars (*Spodoptera exigua*) and sucking aphids (*Myzus persicae*) had significant impacts on plant traits such as plant growth, resource allocation and phytohormone contents. While the changes in plant traits did not affect the overall performance of the root-knot nematodes (*Meloidogyne incognita*) in terms of total number of galls, we found that the consequences of aboveground herbivory for the plants can be altered by the subsequent nematode herbivory. For example, plants that had hosted aphids showed compensatory growth when they were later challenged by nematodes, which was not apparent in plants that had hosted only aphids. In contrast, plants that had been fed by *S. exigua* larvae did not show such compensatory growth even when challenged by nematodes.

**Conclusion:**

The results suggest that the earlier aboveground herbivory can modify plant responses to subsequent herbivores, and such modifications may depend upon identity and/or feeding modes of the aboveground herbivores.

**Electronic supplementary material:**

The online version of this article (doi:10.1186/s12898-017-0115-2) contains supplementary material, which is available to authorized users.

## Background

Plants respond with morphological, physiological and biochemical changes in their resistance and tolerance traits to deal with herbivores and herbivory stress [1–4]. Besides responses in local tissues which are being attacked, herbivory induces numerous changes in more distant systemic tissues, which can cause indirect interactions between spatially, and in some cases, temporally separated herbivores. Thereby, plants can even mediate indirect interactions between phytophagous organisms living above- and belowground [5–10].

Although defensive quality of roots has been analyzed less than that of aboveground plant parts, several plant species are known to systemically induce defensive compounds in roots following aboveground herbivory which may protect them from belowground herbivores [7, 8, 11–13]. Along with chemical defense, plants may also employ tolerance strategies to deal with herbivory, such as altered photosynthetic rates, compensatory growth, increased tillering, and reallocation of primary metabolites and minerals [14, 15]. Plants fine-tune their resistance and tolerance ability in order to optimize plant fitness; therefore, they may or may not employ both strategies simultaneously [12]. Any of the systemic changes in root tissue due to aboveground herbivory, either in resistance or tolerance traits, may significantly impact the performance of subsequent belowground herbivores [16–18]. Recent studies also suggest that sequential herbivory events may result in the priming of plant responses which is a preconditioning by earlier herbivory that enables plants to deal with future herbivores more efficiently [19–21]. Overall, the aboveground herbivore-induced changes in root tissue can be detrimental, neutral or facilitative to the belowground herbivores depending upon several factors such as herbivore species, their feeding guild, plant species, genotypes and defense strategies [7, 16, 22–28].

One of the significant determinants of the outcome of above- and belowground herbivore interactions is the feeding mode of the herbivores. Chewing and sucking are two major feeding modes of herbivores. Coleopteran and lepidopteran insects are equipped with chewing or tearing-type mouthparts causing severe wounding injury whereas hemipteran insects such as aphids and whiteflies are equipped with piercing and sucking mouthparts to ingest the phloem-sap causing minimal injury on plant tissue [29–31]. Wound trauma inflicted by the feeding damage and type of elicitors present in oral secretion of herbivores are two major cues that regulate the induction of specific resistance or tolerance responses of the plant [4, 32]. Therefore, the feeding mode and the identity of the herbivore are key factors in plant–insect interactions as they determine specific activation patterns of plant signaling pathways that regulate a plant’s response. Plant responses upon herbivory are mainly regulated by three phytohormones, jasmonic acid (JA), salicylic acid (SA) and ethylene, which are also known to play essential roles for the growth and development of the plant. A large body of evidences suggests that chewing herbivores primarily activate JA-dependent defense pathways whilst sucking herbivores induce predominantly SA- along with JA- and ethylene-dependent pathways similar to the responses induced by plant pathogenic microbes [29, 33]. But, it is important to note that their activation is highly species-specific and not limited to particular feeding guilds. Several studies have shown the activation of SA-dependent responses upon chewing herbivores and activation of JA-dependent responses upon sucking herbivores; and the phytohormones may interact antagonistically or synergistically with each other [33–35].

Here, we aimed to compare the effects of aboveground herbivory by insects from two feeding guilds (chewing caterpillars and sucking aphids) on plant traits and the plant’s interaction with spatially and temporally separated belowground root-knot nematode. Root-knot nematodes (*Meloidogyne*) are endoparasites which, with the help of special gland secretions, stimulate the root cells to grow into ‘giant cells’ (root-knots or galls) that serve as a feeding site [36]. By inducing galls and feeding on the root tissue, root-knot nematodes weaken the ability of the root to take up water and nutrients which impairs plant performance and fitness [37]. Although nematodes do not feed by sucking up phloem sap like aphids, their feeding strategies and salivary composition have noticeable similarities [38, 39] and both are sensitive to plant resistance traits mediated by the same gene, *Mi*-*1* [40] which is found in tomato (*Solanum lycopersicum*). Commercial tomato cultivars are known to contain the *Mi* locus with two highly homologous genes, *Mi*-*1.1* and *Mi*-*1.2* [37] which confer resistance against aphids [40, 41], whiteflies [42] and root-knot nematodes including *Meloidogyne incognita* [37, 43]. Furthermore, subsequent studies found that the SA signaling pathway is essential for *Mi*-*1*-mediated defense responses, suggesting its inducibility [44, 45]. Therefore, aphids, nematodes and tomato plants are an interesting model system to study plant-mediated impacts of aboveground herbivores on belowground herbivores. In our study, we hypothesized that earlier transient aboveground herbivory by aphids would have a more pronounced impact on nematodes because of activation of the same defense pathway than transient chewing herbivory by caterpillars.

To differentiate between the effects of plant responses to herbivores of different feeding modes on the plant’s interaction with root-knot nematodes (*M. incognita*, Heteroderidae), we used the sap-feeding green peach aphid (*Myzus persicae*, Aphididae) and the chewing beet armyworm (*Spodoptera exigua*, Noctuidae). Using tomato (*S. lycopersicum,* Solanaceae var. MicroTom) as a model plant, we aimed to investigate: (1) if transient aboveground herbivory has any effect on plant traits and affects spatially and temporally separated belowground herbivores; (2) if transient aboveground herbivory affects the plant's response to the subsequent belowground herbivory; (3) if those effects differ between the two aboveground herbivore species exhibiting different feeding modes. To answer these questions, we carried out a greenhouse experiment in which tomato plants were exposed to transient herbivory by either aphids, caterpillars or no aboveground herbivores, followed by nematode infestation or not. We separated the events of above- and belowground herbivory by a lag phase (a period without any herbivory) to assess the effect of transient aboveground herbivory on temporally separated belowground herbivores.

## Methods

### Plant material

Before germination, the seeds of tomato (*S. lycopersicum*) were surface-sterilized with 70% ethanol followed by mixture of 5.25% (w/v) sodium hypochlorite and 0.1% Polysorbate 20 (Tween 20). Then, the seeds were rinsed with deionized water and sown on paper towels in plastic boxes and kept in the greenhouse at 26 °C for a week to germinate. The seedlings of about 2 cm height were transplanted to seedling trays for a month before finally being transferred to 1 l (13 × 11 × 9 cm^3^) plastic pots (Pöppelmann GmbH & Co. KG, Lohne, Germany) containing 850 ml of steamed soil. The soil was collected from a research site of Freie Universität Berlin (Albrecht-Thaer-Weg) and sieved to remove the remains of roots and pebbles. The sieved soil was steamed for three hours at 90 °C using a Sterilo steamer (Harter Elektrotechnik, Schenkenzell, Germany) to exclude root herbivores. Pots were placed on individual plastic plates and the top layer of the soil was covered with sand grit to prevent the growth of green algae and infestation by fungus gnats (Sciaridae). The plants were assigned to different treatments after 3 weeks of growth in pots. During the experiment, plants were watered three times a week with 150 ml of water and randomized weekly to homogenize for variances due to abiotic factors such as light conditions.

### Study insects

The green peach aphid (*M. persicae*) individuals used in this experiment were obtained from the aphid rearing of the Julius Kühn-Institute, Berlin. The larvae of beet armyworm *S. exigua* were obtained from the laboratory cultures maintained at the Freie Universität Berlin. They were reared on artificial diet (wheat germ based basic diet with a vitamin mix) in a climate chamber at 24 °C and 70% humidity under 16/8 h day/night light cycle. Second-stage juveniles (J2s) of root-knot nematodes *M. incognita* were obtained in aqueous suspension from a biological supply company, HZPC Holland B. V. (Hettema Zaaizaad en Pootgoed Coöperatie, Metslawier, The Netherlands).

### Herbivory treatments

For the herbivory treatments, a total of 90 healthy and homogeneous plants were selected. Plants were subjected to six different treatments with 15 replicates each: control with no herbivory (C), aboveground herbivory with *M. persicae* aphids (Aph) or *S. exigua* larvae (Spo), belowground herbivory with *M. incognita* nematodes (Nem), and sequential above- and belowground herbivory treatments (Aph + Nem and Spo + Nem) where nematodes were added to the root of the aboveground herbivore-treated plants following a lag phase of seven days. For the aboveground herbivory treatments, the three youngest, fully expanded leaves were chosen on every plant. In the treatments with the chewing herbivore, one third instar *S. exigua* larva was added in a mesh bag and allowed to feed on the first leaf for three days starting with the oldest among the three chosen leaves. The larva was then transferred successively to the second and the third leaf to feed for another two days on each. This way, larvae fed on three consecutive leaves for a total of seven days. In the treatments with the sucking herbivore, four individuals of *M. persicae* were added on each of the three leaves which were covered with a mesh bag. Aphids were allowed to feed on leaves for seven days and then removed carefully using a fine brush without damaging the leaves. After the removal of aboveground herbivores, the plants were kept for a lag phase of seven days without herbivory. Then, about 1875s stage juveniles (J2′s) of root-knot nematodes *M. incognita* were added per pot as belowground herbivore to the roots of half of the aboveground herbivore-treated and half of the control plants. The nematodes were applied in an aqueous suspension in three holes (depth 5 cm) perforated into the soil at a distance of 3 cm from the stem. These plants were treated for 14 days with the nematodes allowing them to infest the roots and induce root galls before harvest. Upon harvest, leaf and root subsamples were collected for the phytohormone analysis. The numbers of galls induced by the nematodes were counted in three different size classes (<1, 1–2 and >2 mm) manually after keeping them submerged in water to facilitate the counting. The root (including galls) and shoot materials were then dried in an oven at 55 °C for three days before measuring the dry mass.

### Sampling and measurement of phytohormone

For the phytohormone measurement, the roots of the harvested plants were washed immediately after harvest and 150–180 mg of representative fine root samples were separated and weighed. A similar amount was also collected of leaf samples from the youngest fully expanded leaf by cutting it transversely into small pieces. The leaf and root samples were kept in 2 mL screw-cap tubes, flash frozen in liquid nitrogen and stored at −80 °C until extraction. Extraction and quantification of ABA, SA, JA and JA-isoleucine (JA-Ile) were done following the procedure explained in [46]. In brief, root and leaf samples were homogenized within the tubes using FastPrep homogenizer (FastPrep^®^-24, MP Biomedicals, Santa Ana, CA, USA) along with 1 ml extraction solution, containing ethyl-acetate and internal deuterated standard mix: 20 ng of D4-SA, D6-ABA (OlChemIm Ltd., Olomouc, Czech Republic) and D6-JA-Ile and 60.4 ng of D6-JA (HPC Standards GmbH, Cunnersdorf, Germany). Supernatant was collected after centrifuging the homogenized samples for 5 min at high speed (18,000×*g*). Samples were extracted a second time with 1 mL pure ethyl-acetate, then supernatants were combined and dried in a Vacufuge (Eppendorf, Hamburg, Germany). The dried samples were re-eluted in 400 μL of 70% (v/v) methanol (MeOH) and 0.1% acetic acid by shaking 10 min at room temperature. The re-eluted extracts were subjected to a UPLC-ESI–MS/MS Synapt G2-S HDMS (Waters, Milford, Massachusetts, USA) for identification and quantification of phytohormones as described in [46]. The peak area integration was performed using MassLynx Software v. 4.1 (Waters, Milford, Massachusetts, USA). The amount of hormone per g of sample fresh weight was calculated by comparing the peak area of the plant derived hormone in a given sample with the corresponding peak area of the deuterated internal standard in the same sample. From the pool of 15 replicates, eight replicates from each treatment were chosen randomly for hormonal measurement.

### Carbon and nitrogen concentration measurement

Dried leaf and root materials were ground in Eppendorf tubes by using a mixer mill (Mixer Mill MM 400, Retsch GmbH, Haan, Germany) and dried again for at least 24 h. Then, their carbon and nitrogen concentration were determined by using an elemental analyzer (Euro EA, HEKAtech GmbH, Wegberg, Germany).

### Statistical analysis

All the statistical analyses were performed in ‘R’, version 3.2.2 [47]. One-way and two-way factorial ANOVAs were performed to test the significance of the treatments; aboveground herbivory (AGH), belowground herbivory (BGH) and their interactions (AGH*BGH). Statistical significance was set at *P* < 0.05. All the data were checked for normality and homogeneity of variances using Shapiro–Wilk test and Bartlett test, respectively, to make sure that they meet the assumptions of ANOVA. The data of number of galls and root C concentration were transformed using log and square transformation, respectively, while the data of shoot biomass and root C/N ratio were transformed using inverse transformation before being checked for assumptions of ANOVA. The phytohormone data were analyzed with Generalized Linear Models (GLM) assuming gamma distribution of errors as the data were not normally distributed. Means and standard errors (SE) are reported in the result section. To determine the effects of the particular aboveground herbivores, the means were additionally compared with Tukey HSD test as post hoc analysis.

## Results

### Plant biomass

**Fig. 1:**
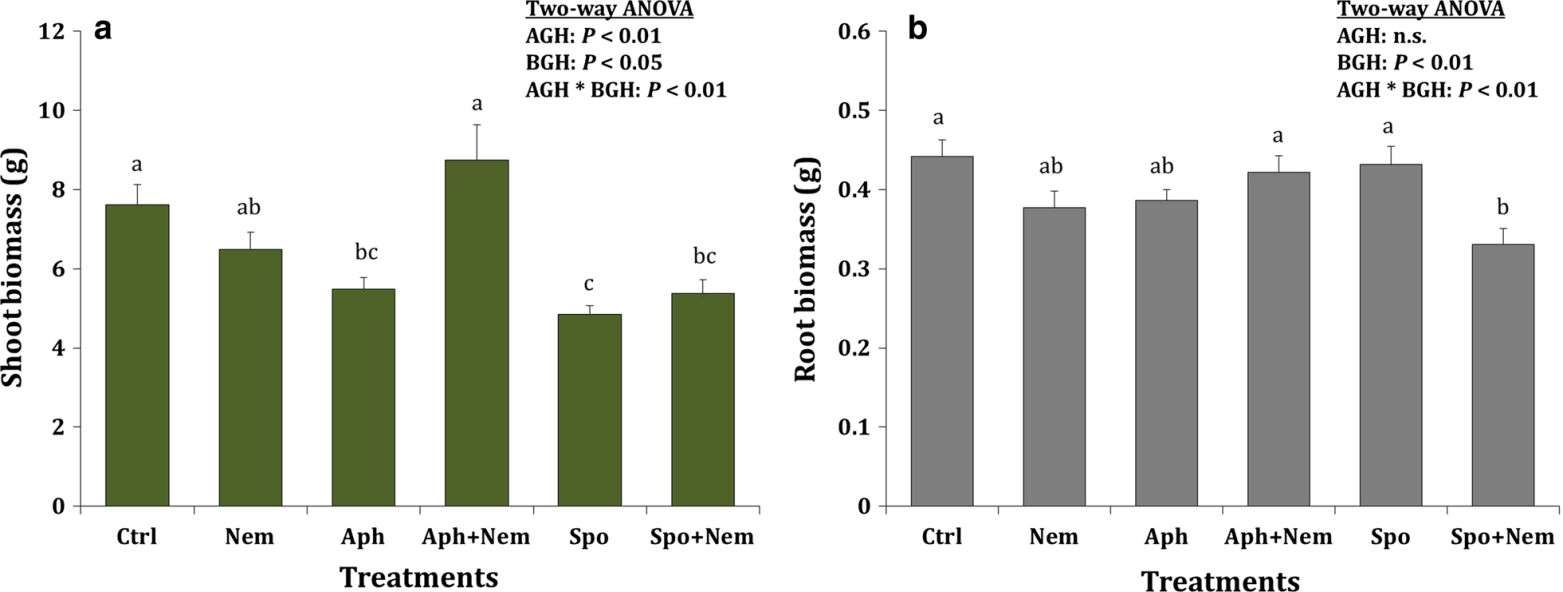
Shoot (**a**) and root (**b**) biomass (mean ± SE; n = 15) of the tomato plants following herbivory treatments. *Treatments*: Ctrl: control (no herbivory), *Nem* nematode only, *Aph* aphids only, *Aph* *+* *Nem* aphids followed by nematodes, *Spo S. exigua* larvae only, *Spo* *+* *Nem S. exigua* larvae followed by nematodes. *Different letters* above the *bar* indicate the significant difference in their mean. Aboveground herbivory (AGH) was applied for a week and belowground herbivory (BGH) was applied for two weeks while there was a lag phase of a week between AGH and BGH in sequential herbivory treatments

Shoot biomass: Both above- and belowground herbivory had significant main and interaction effects on shoot biomass (AGH: F_[2, 84]_ = 16.58, *p* = 0.001; BGH: F_[1, 84]_ = 5.05; *p* = 0.027; AGH*BGH: F_[2, 84]_ = 9.08; *p* < 0.001). When applied alone, both aphid and *S. exigua* herbivory reduced the shoot biomass. The negative effect of *S. exigua* remained stable under single or sequential herbivory exposure; the negative effect of aphid herbivory was abolished when followed by nematode infestation although nematode infestation alone did not significantly affect shoot biomass (Fig. 1a).

Root biomass: Aboveground herbivory had no significant main effect on root biomass, while belowground herbivory significantly reduced root biomass, which was significantly affected by the interaction with aboveground herbivory (AGH: F_[2, 84]_ = 1.13, *p* = 0.33; BGH: F_[1, 84]_ = 7.07; *p* = 0.001; AGH*BGH: F_[2, 84]_ = 6.20; *p* = 0.003). Earlier *S. exigua* herbivory followed by the nematode treatment reduced the root biomass by about 25% as compared to *S. exigua* alone and control plants; aphids and nematodes alone and in combination did not significantly differ from the control (Fig. 1b).

### Carbon and nitrogen concentration

We measured the changes in C and N concentration in leaf and root tissue following herbivory to estimate changes in allocation of these major constituents of plant metabolites and because plant as well as herbivore performance parameters are known to depend on C/N contents.

Leaf C and N concentration: None of the herbivory treatments had any significant effect on the foliar C concentration. Aboveground herbivory had a significant main effect on leaf N concentration and a significant interaction effect with belowground herbivory as *S. exigua* feeding increased foliar N which was stronger and only significant when its herbivory was followed by nematode infestation. Belowground herbivory alone had no effect on foliar N concentration (Table 1).

**Table 1:** The effect of above- and belowground herbivory treatments on C and N concentration (percentage) and their ratios in leaves and roots of the tomato plants

Tissue	Concentration (Mean ± SE; n = 15)					
	Ctrl	Nem	Aph	Aph + Nem	Spo	Spo + Nem
*Leaf*						
C	38.99 ± 0.36^a^	38.60 ± 0.38^a^	38.66 ± 0.43^a^	38.37 ± 0.5^a^	38.61 ± 0.38^a^	38.56 ± 0.35^a^
N	2.89 ± 0.08^b^	2.92 ± 0.10^b^	2.98 ± 0.08^ab^	2.75 ± 0.04^b^	3.03 ± 0.09^ab^	3.27 ± 0.08^a^
C/N	13.63 ± 0.41^a^	13.41 ± 0.41^a^	13.11 ± 0.38^ab^	14.00 ± 0.25^a^	12.94 ± 0.41^ab^	11.88 ± 0.29^b^
*Root*						
C	41.86 ± 0.56^ab^	43.37 ± 0.42^a^	41.06 ± 0.69^b^	43.05 ± 0.43^ab^	37.94 ± 0.51^c^	42.64 ± 0.56^ab^
N	2.46 ± 0.08^b^	3.02 ± 0.04^a^	2.61 ± 0.07^b^	2.68 ± 0.06^b^	2.53 ± 0.06^b^	3.08 ± 0.05^a^
C/N	17.18 ± 0.48^a^	14.38 ± 0.21^cd^	15.81 ± 0.36^abc^	16.15 ± 0.37^ab^	15.1 ± 0.39^bcd^	13.88 ± 0.21^d^

	ANOVA results					
	AGH		BGH		AGH:BGH	
	*F*	*P*	*F*	*P*	*F*	*P*
*Leaf*						
C	0.267	0.766	0.542	0.464	0.094	0.911
N	7.127	*0.001*	0.047	0.8281	4.279	*0.017*
C/N	6.418	*0.003*	0.194	0.6606	3.56	*0.033*
*Root*						
C	9.706	*<0.001*	38.31	*<0.001*	4.751	*0.011*
N	3.28	*0.042*	61.39	*<0.001*	10.45	*<0.001*
C/N	12.58	*<0.001*	20.04	*<0.001*	9.95	*<0.001*

*Treatments*: Ctrl: control (no herbivory), *Nem* nematode only, *Aph* aphids only, *Aph* *+* *Nem* aphids followed by nematodes, *Spo S. exigua* larvae only, *Spo* + *Nem S. exigua* larvae followed by nematodes. AGH and BGH stand for above- and belowground herbivory respectively. (AGH. *df*: 2, 84; BGH. *df*: 1, 84; AGH:BGH. *df*: 2, 84). Italic fonts indicate the significant effects (P < 0.05) of the treatments. Mean ± SE followed by different letters are significantly different from each other (Tukey HSD test: P < 0.05)

Root C and N concentration: Both above- and belowground herbivores had main and interaction effect in root C and N concentration (Table 1). The *S. exigua* herbivory reduced the C concentrations in the root tissues, while the nematode treatment after *S. exigua* herbivory abolished this effect. Nematodes alone increased root N concentration compared to control plants. This effect was still present in plants previously damaged by *S. exigua*, but the nematodes had no effect on root N concentration if plant were fed by aphids earlier. Aphids or *S. exigua* alone had no effect on root N concentration.

C/N ratio: As the C concentration was similar in all treatments; the change in leaf C/N ratio was dependent on changes in leaf N concentration and therefore had similar patterns as leaf N concentration (Table 1). Plants treated with *S. exigua* followed by nematode decreased the C/N ratio of the leaves but these herbivores alone had no effects. Similarly, there were significant main and interactive effects of both above- and belowground herbivores on the C/N ratio of the roots. Single herbivory by *S. exigua* and nematode, and sequential herbivory by *S. exigua* followed by nematodes decreased the C/N ratio in the roots as compared to control plants.

### Phytohormone induction

**Fig. 2:**
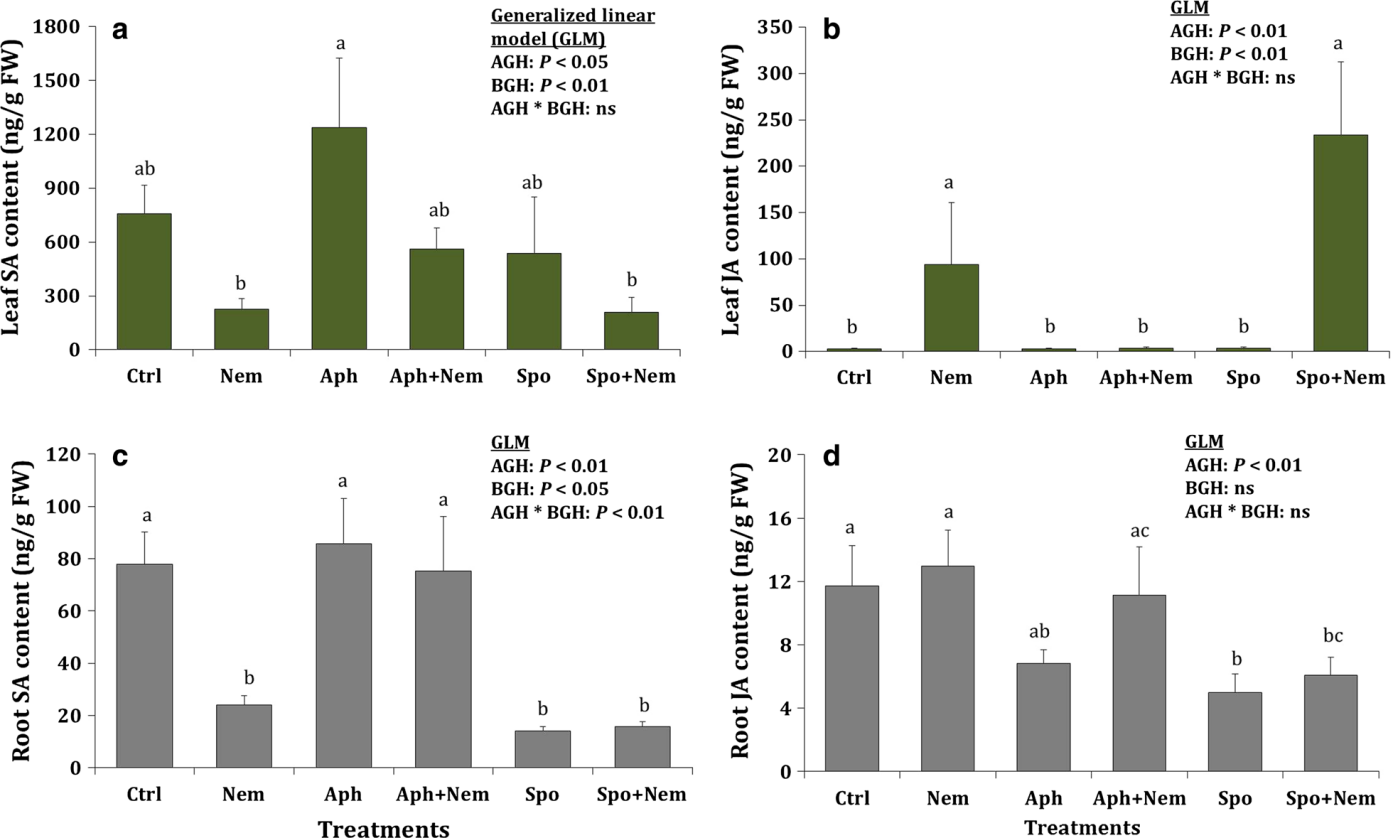
Leaf (**a**, **b**) and root (**c**, **d**) SA and JA content of the tomato plants (mean ± SE; n = 8) following herbivory treatments. *Treatments*: Ctrl: control (no herbivory), *Nem* nematode only, *Aph* aphids only, *Aph* *+* *Nem* aphids followed by nematodes, *Spo S. exigua* larvae only, *Spo* *+* *Nem S. exigua* larvae followed by nematodes. *Different letters* above the *bar* indicate the significant difference in their mean. Aboveground herbivory (AGH) was applied for a week and belowground herbivory (BGH) was applied for two weeks while there was a lag phase of a week between AGH and BGH in sequential herbivory treatments

There were significant main effects of the above- and belowground herbivores on both salicylic acid (SA) and jasmonic acid (JA) content of the leaf tissues at a time point that was three weeks after the aboveground herbivory and after two weeks of exposure to nematodes. Nematodes had a significant negative main effect on leaf SA content (Fig. 2a), while nematodes either alone or following *S. exigua* herbivory increased the leaf JA content, which did not occur on plants previously infested with aphids (Fig. 2b). Above- and belowground herbivores had significant main effects and interaction effects on root SA content, while the root JA content was affected by the aboveground herbivores only. Whereas nematodes and *S. exigua* alone and in combination significantly reduced SA contents in the roots, previous aphid herbivory abolished this effect of nematodes on root SA (Fig. 2c). On the other hand, *S. exigua* larvae alone or followed by nematodes decreased root JA content compared to control plants (Fig. 2d).

### Number of galls induced by nematodes

The total number of galls and number of galls per mg of root tissue induced by nematodes did not differ between the treatments. There was a significant reduction of the number of small galls per mg of root tissue (<1 mm) in the plants previously treated with aphids compared to plants treated with nematodes only (*p* = 0.01) while total number of small galls (not corrected for root mass) tended to be reduced (*p* = 0.07) (Additional file 1).

## Discussion

Our study demonstrated that the transient aboveground herbivory by both chewing and sucking herbivores had significant impact on root and shoot parameters, nutrient allocation and the activation of signaling components (phytohormones). The consequences of transient aboveground herbivory on plant traits had no major effect on overall nematode performance (in terms of total number of galls), but plants previously exposed to aphids showed a reduced number of small galls per unit root mass. Transient aboveground herbivory changed the plant response to the later root infestation by nematodes. The way in which the plant response was altered by the sequential herbivory, was different for the two aboveground herbivores highlighting the significance of the herbivores’ identities. As the two herbivores used in this experiment exhibit different feeding modes, the plants’ distinct response to them could in part have resulted from the different feeding modes [29, 33].

### Effect of transient aboveground herbivory on belowground herbivores

A recent meta-analysis suggests that the aboveground herbivore, if it arrives first on the plant, is expected to have negative effects on the performance of belowground herbivores [26]; however we found no negative effects of aboveground herbivores on the overall performance of nematodes in terms of total number of galls. For example, an experiment [24] with cultivated and wild maize plants (*Zea mays mays* and *Z. mays mexicana*) showed that feeding by the aboveground chewing herbivore *Spodoptera frugiperda* had a significant negative effect on the root chewing herbivore *Diabrotica virgifera* in terms of root colonization and weight gain, but only if *S. frugiperda* was added first to the plant. Thus, we expected an overall negative effect of aboveground herbivores, which were added first on the plant, on the belowground herbivore. Additionally, we expected even stronger responses of the plants treated first with aphids, as tomato plants are known to respond with a similar arsenal of defenses against aphids and nematodes, namely the *Mi*-*1* gene dependent defense responses which require SA signaling [44, 45]. However, although *Mi*-*1* gene was found in commercial tomatoes, some tomato varieties lack it [44] and it remains unclear whether the MicroTom cultivar contains it. While we did not find elevated SA levels in roots or shoots of plants that had been exposed to aphids three weeks earlier, we found reduced levels of root SA in nematode-infested plants and in plants that had been attacked by *S. exigua* and nematodes. The negative effect of nematodes and *S. exigua* on root SA which likely resulted in a reduced root resistance due to a lack of SA-mediated defense was absent in plants with earlier transient aphid herbivory. The plants previously attacked by aphids showed no reduction in root SA after nematode herbivory, and such plants had a reduced number of smaller galls. The finding of a negative effect of nematodes on root SA that was negated on plants with earlier aphid herbivory highlights the effect of earlier aboveground herbivory and their identity on plant response to subsequent belowground herbivores.

### Plant response upon above- and belowground herbivory

Both above- and belowground herbivores had significant effects on plant growth that differed in direction and magnitude. While *S. exigua* herbivory reduced the shoot biomass independent of a later nematode infestation, the negative effect of aphid herbivory on shoot biomass was abolished, when subsequently nematodes fed on the same plants. This suggests that aphid-treated plants showed a compensatory growth of shoots upon nematode herbivory, while *S. exigua* larvae-treated plants did not compensate for the loss in biomass upon nematode herbivory. Nematode addition may have facilitated a tolerance response of the tomato plants such as a compensatory growth to replenish the biomass loss due to aphid herbivory. On the other hand, nematode infestation or *S. exigua* herbivory alone had no significant effects on root biomass, whereas these herbivores in sequential combination reduced the root biomass. This result further demonstrates the altered plant response to subsequent belowground herbivores due to their earlier exposure to aboveground herbivores. Such reduction in root biomass was not evident in the plants treated with aphids followed by nematodes suggesting the significance of aboveground herbivore identity.

Regarding the allocation pattern of C and N in leaf and root tissues, most noticeable effects were found in the N concentration of the plants subjected to sequential above- and belowground herbivory: plants previously exposed to *S. exigua* and followed by nematode infestation contained higher N concentration in both leaf and root tissue. In root tissue, nematodes also increased the N concentration but not when the plants had been previously exposed to aphid feeding. Interestingly, the direction of change in N concentration was opposite to the changes in biomass of the plants infested by *S. exigua* followed by nematode. These results suggest that the nutritional quality may be improved in the shoot and root tissues of the plants whose biomass was decreased in the *S. exigua* followed by the nematode treatment. Systemic nutrient translocation to and away from the site of herbivory is another well-known tolerance response of the plants upon herbivory. Plants allocate carbon and nitrogen in specific cells and tissues to be used for compensatory growth or defense of valuable plant parts which are critical for survival and reproduction [48]. In addition, such diversion of nutrients results in poor nutritional quality of the feeding site with possible negative effects on growth of herbivores [3, 16, 49–51]. Further, increased N in both shoot and root tissues in plants treated with *S. exigua* and later with nematodes may indicate the acquisition of additional N from the soil pool to meet the increased demand of N for either compensatory growth or for biosynthesis of N-based defense compounds such as protease inhibitors (PIs). However, such potential increase in N compounds did not contribute to resistance against nematodes. On the contrary, increased N may also promote herbivore performance by enhancing the nutritional quality of plant tissue.

We measured the defense-regulatory phytohormones JA and SA which may allow some estimation on the level of induced defense in the leaf and root tissue upon sequential above- and belowground herbivory. The defensive functions of SA and JA in tomato against herbivores has been studied in detail in previous studies. For example, SA was found to be an essential component of the *Mi*-*1* mediated resistance against both aphid and nematode in tomato plants [44, 45]. An earlier study [52] has demonstrated that the JA is also an essential and dominant regulatory component for the induction of not only direct plant defense compounds such as polyphenol oxidases (PPOs) but also indirect plant defense compounds such as volatiles. In addition, defense signaling pathways mediated by these phytohormones are known to coordinate with several other pathways in a complex regulatory network that governs growth and defense physiology of plants and understanding the role of each of such pathways is still a challenge in ecological studies. We found herbivore-specific alterations of phytohormone levels in both leaf and root tissues. Nematode herbivory increased the leaf JA content but not on plants that had been previously exposed to aphids, whereas prior *S. exigua* herbivory did not alter this JA-induction by nematodes. The roots of plants previously attacked by caterpillars had lower JA levels independent of a later nematode infestation. On the other hand, both *S. exigua* and nematode herbivory either alone or in combination decreased the root SA content, while previous aphid herbivory reversed the negative effect of nematodes on root SA, which might be related to the lower number of small nematode galls per root mass in previously aphid infested plants. Although speculative, this finding may indicate an increased nematode resistance of plants upon aphid exposure due to stronger SA-mediated defenses, which would be in line with the concept of defense priming [19, 53]. However, whether a priming of plant defense is involved in the interactions between above- and belowground herbivory that we determined would require further investigation. In general, plant defense is considered to be costly for example in terms of resources that are required for the production of defense compounds [54]. And if the costs of defense outweigh the cost of herbivory, plants may employ other strategies such as tolerance which is an alternative plant strategy to cope with herbivory stress [15]. In our study, tomato plants were able to compensate for the loss of shoot biomass due to aphid herbivory when they were later exposed to nematodes indicating a tolerance response that is only triggered by the sequential herbivory.

### Role of herbivores’ identity and feeding mode in plant–insect interaction

As we hypothesized, herbivore identity was a key factor to bring specific changes in plant traits. All the changes in measured parameters such as biomass, C and N distribution and phytohormone content in both leaf and root tissue upon nematode herbivory were dependent on the identity of the shoot herbivores. For example, plants previously treated with *S. exigua* herbivory contained higher N concentration in both leaf and root tissue upon nematode infestation, while previous aphid feeding had no such effect. There is some evidence that the induced response of tomato differs upon herbivory by insects of different feeding guilds; for example, aphid (*Macrosiphum euphorbiae*) feeding was found to induce peroxidase and lipoxygenase, but not PPO and PIs, while noctuid insect *Helicoverpa zea* feeding induced PPO, PIs, and lipoxygenase, but not peroxidase [55]. Similarly, another study [56] showed that herbivory by *S. exigua* increased the PI activity by three times as compared to control plants, whereas aphid (*M. euphorbiae*) herbivory did not induce such effects in tomato plants. For the efficient use of limited resources, plants respond to herbivores by activating a specific array of resistance and tolerance to deter herbivores which share similar characteristics such as feeding mode. Therefore, such specific defense strategies targeted at herbivores with different feeding modes might explain the differences we find in the plant response to sequential attack by aphids, caterpillars and nematodes.

## Conclusion

In summary, our study showed for the first time that transient aboveground herbivory modified the plant response to subsequent root herbivory, and herbivores’ identity and probably the feeding mode of the aboveground attacker had significant influence on such modification. Although earlier transient herbivory had no detrimental effect on the overall performance of belowground herbivore, the plant responded with compensatory shoot growth to sequential aphid and nematode herbivory. Herbivore-induced plant responses such as compensatory growth and root exudation may affect species across different trophic levels which may eventually affect species composition and diversity in terrestrial ecosystems [9, 57]. Our study provides a small glimpse on the complexity of plant-herbivore interactions and shows that it is important to study interactions between multiple organisms above- and belowground to complement our understanding of plant-herbivore ecology.

## Additional file

**Additional file 1.** Number of root galls (knots) induced by the nematodes.
