# Supplementary material for: Sequential above- and belowground herbivory modifies plant responses depending on herbivore identity
Source: BMC Ecol. 2017 Feb 8;17:5. doi: 10.1186/s12898-017-0115-2 (PMC5299658; doi:10.1186/s12898-017-0115-2)
Supplement: Supplementary file 1 — Additional file 1. Number of root galls (knots) induced by the nematodes. [file 12898_2017_115_MOESM1_ESM.pdf]

Additional fig. 1:

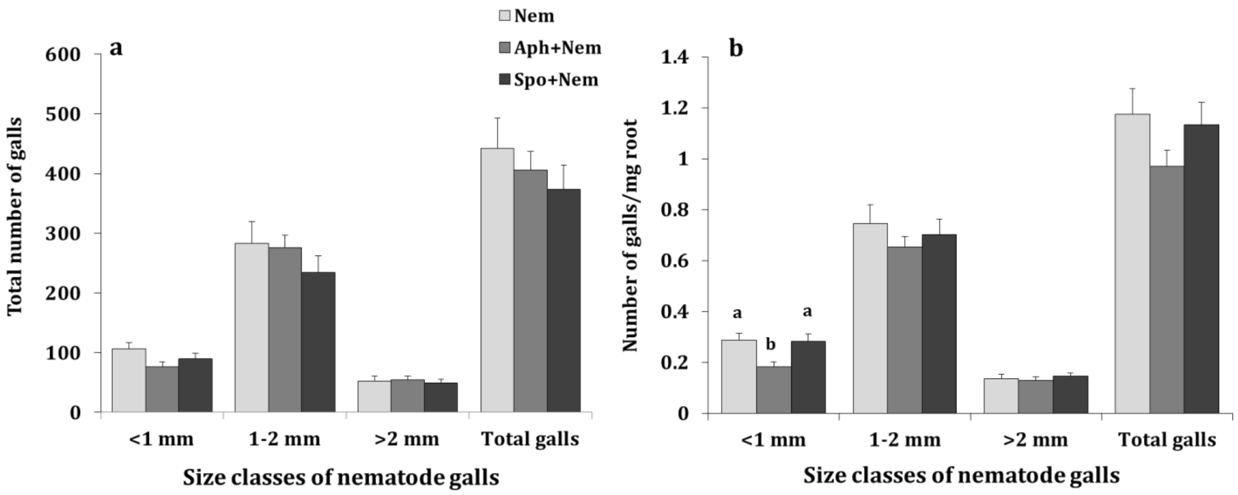

Additional fig. 1. Total number of galls of different size classes (mean ± SE; n=15) (a) and number of galls of different size classes per mg of roots (b) of tomato plants treated only with nematodes (Nem), treated with aphids followed by nematodes (Aph+Nem) and treated with *S. exigua* larvae followed by nematodes (Spo+Nem). Aboveground herbivory was applied for a week and nematode herbivory was applied for two weeks while there was a lag phase of a week between above- and belowground herbivory in sequential herbivory treatments. Different letters above the bar indicate the significant difference in their mean.
